# *In vitro* developmental ability of ovine oocytes following intracytoplasmic injection with freeze-dried spermatozoa

**DOI:** 10.1038/s41598-017-00583-0

**Published:** 2017-04-24

**Authors:** Maite Olaciregui, Victoria Luño, Paula Domingo, Noelia González, Lydia Gil

**Affiliations:** Animal Pathology Department of Veterinary Faculty, Universidad de Zaragoza, Miguel Servet 177, 50013 Zaragoza, Spain

## Abstract

Freeze-drying (FD) is a new and alternative method to preserve spermatozoa in refrigeration or at room temperature. Suitable protection is required to maintain the sperm DNA integrity during the whole process and storage. The aim of this study was to examine the effect of rosmarinic acid and storage temperature on the DNA integrity of freeze-dried ram sperm. In addition, we evaluated the *in vitro* developmental ability to the blastocyst stage of oocytes injected with freeze-dried sperm. Ram sperm was freeze-dried in basic medium and in this medium supplemented with 105 µM rosmarinic acid. The vials were stored for 1 year at 4 °C and at room temperature. Frozen sperm was used as control. After rehydration, sperm DNA damage was evaluated, observing that the percentage of spermatozoa with DNA damage decreased significantly in the presence of rosmarinic acid, without differences between the two storage temperatures. Moreover, no differences were observed between the freeze-dried group and the frozen-thawed group in terms of blastocyst formation rate. We proved for the first time that ovine spermatozoa can be lyophilized effectively, stored at room temperature for long term, reconstituted and further injected into oocytes with initial embryo development.

## Introduction

Freeze-drying sperm preservation is a new and attractive method by which spermatozoa can be long-term stored both in a refrigerator and at room temperature. That means that no liquid nitrogen is required, which considerably reduces the costs of doses storage and shipping. Nowadays, this method has been applied to different domestic species, such as rabbit^1^, horse^2, 3^, cat^4^, dog^5, 6^, boar^7^ and bull^8^. However, we did not find any study in which this technique had already been applied on ram sperm.

In spite of the advantages of this method, the main limitation is still the damage caused on the sperm. In particular, the spermatozoa lose their motility after freeze-drying and intracytoplasmic sperm injection (ICSI) is needed to fertilize oocytes^9^. Besides, sperm DNA might be damaged by the action of DNAases or oxidative stress during the freezing and freeze-drying processes. Kusakabe *et al*.^10^ suggested that the release of endogenous nucleases from the plasma membrane of damaged spermatozoa after sperm freeze-drying is the most likely reason to produce structural chromosome aberrations. They found that chromosome integrity of mouse spermatozoa can be maintained during freeze-drying when the spermatozoa are suspended in a simple Tris-HCl-buffered solution containing 50 mM EGTA and 50 mM NaCl.

On the other hand, oxidative stress might represent another mechanism of damage, leading to spermatozoa DNA fragmentation during freeze-drying and rehydration procedures^10, 11^. Exposure of DNA to ROS (reactive oxygen species) induces a wide variety of chemical changes that include cross-linking, base modification and DNA strand breakage^12^. It has been already reported that the ram is one of the species with the fastest sperm DNA degradation^13^. The beneficial effect of antioxidant therapy on the oxidative stress in mammalian spermatozoa has widely been studied^14–19^. It has been well characterized that the addition of antioxidants to freezing extenders decreases the detrimental effects of ROS^20^. Rosmarinic acid (RA) is a natural antioxidant that protects cells from oxidative stress *in vitro* ^21^. It has been proved that the incorporation of RA in freezing extenders improved sperm quality after cryopreservation^20^. However, to our knowledge, no studies have been performed to evaluate the effect of antioxidants on freeze-dried ram sperm.

Furthermore, the DNA of the freeze-dried sperm can also be damaged if the adequate protection is not provided during the storage^22^. Preservation at a low temperature is important for a stable long-term storage of freeze-dried spermatozoa, being 4 °C the most suitable temperature for this purpose^23, 24^. Nevertheless, storage at room temperature would be ideal and would allow a simple transport of freeze-dried samples around the world^25^. Kusakabe *et al*.^10, 11^ already determined that freeze-dried samples can be transported for long distances at ambient temperatures without any consequence regarding chromosomal damage or loss of developmental potential. However, it is still required to further develop new strategies in order to be able to transport and to preserve indefinitely sperm samples with no impact on sperm quality and excluding liquid nitrogen or dry ice.

The aim of this study was to examine the effect of rosmarinic acid and the influence of the storage temperature on the DNA integrity of freeze-dried ram sperm. In addition, we evaluated the *in vitro* developmental ability to the blastocyst stage of injected ovine oocytes with freeze-dried ram sperm.

## Results

### Effect of rosmarinic acid and storage temperature on DNA integrity

**Figure 1:**
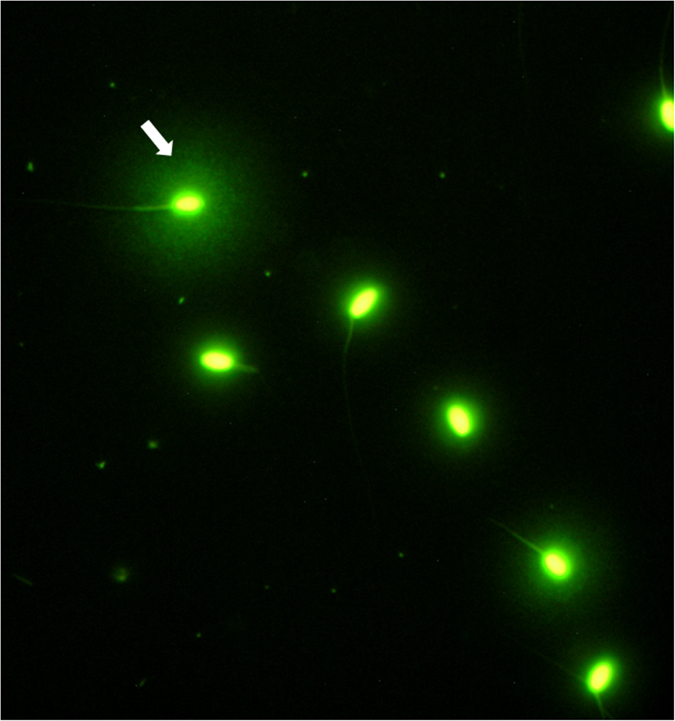
SCD-processed freeze-dried sperm sample with Syber-Green fluorescence: sperm nuclei with fragmented DNA exhibit a large and spotty halo of chromatin dispersion (arrow). Sperm nuclei that exhibited small and compact halos of chromatin dispersion corresponded to spermatozoa with unfragmented DNA.

**Figure 2:**
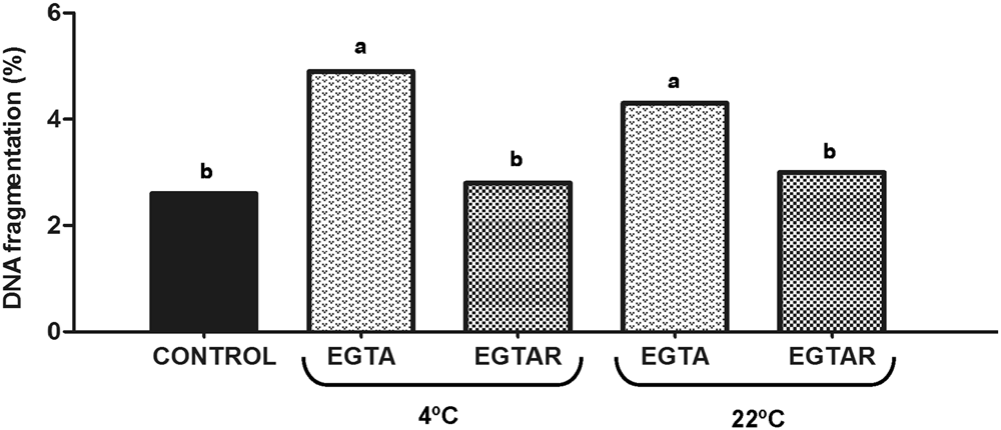
Effects of rosmarinic acid and storage temperature on DNA integrity of freeze-dried ram sperm. Sperm DNA fragmentation was assessed by Sperm cromatin dispersion test (SCD). Five replicated trials were carried out for each group and a minimum of 300 spermatozoa were counted per semen sample. Different letters indicate significant differences at the P < 0.05 level, among the four treatments studied.

Halomax reflected the DNA fragmentation in each sperm head; the bigger size of halos indicated the more accelerated level of DNA fragmentation (Fig. 1). The results obtained by using the sperm chromatin dispersion test to analyze the DNA integrity of the freeze-dried ram sperm are showed in Fig. 2. The sperm samples evaluated after thawing (control) showed higher percentage of sperm with intact DNA (97.4%) than sperm freeze-dried with EGTA without rosmarinic acid supplementation. Regardless of the storage temperature, when RA was added to freeze-drying medium the percentage of spermatozoa with DNA damage decrease significantly (p = 0.003). The spermatozoa lyophilized with EGTAR had 2.9% chromatin damaged cells (large and spotty halo) whereas sperm lyophilized with EGTA had 4.4% chromatin damaged cells. For both storage temperatures, 4 °C (p = 0.004) and 22 °C (p = 0.032), the DNA damage levels in EGTA group were higher than those in EGTAR and control groups.

When the temperature of storage was analyzed, there were no significant differences between the two temperatures studied (p = 0.269). Regardless of the freeze-drying medium used, the percentage of sperm with intact DNA no differed when they were storage at 4 °C (96.2%) or at room temperature (96.5%).

Freeze-dried sperm in both treatments and stored at room temperature were used for injection into IVM oocytes for examination of subsequent *in vitro* developmental competence.

### Embryonic development obtained after ICSI with frozen-thawed or lyophilized spermatozoa

**Figure 3:**
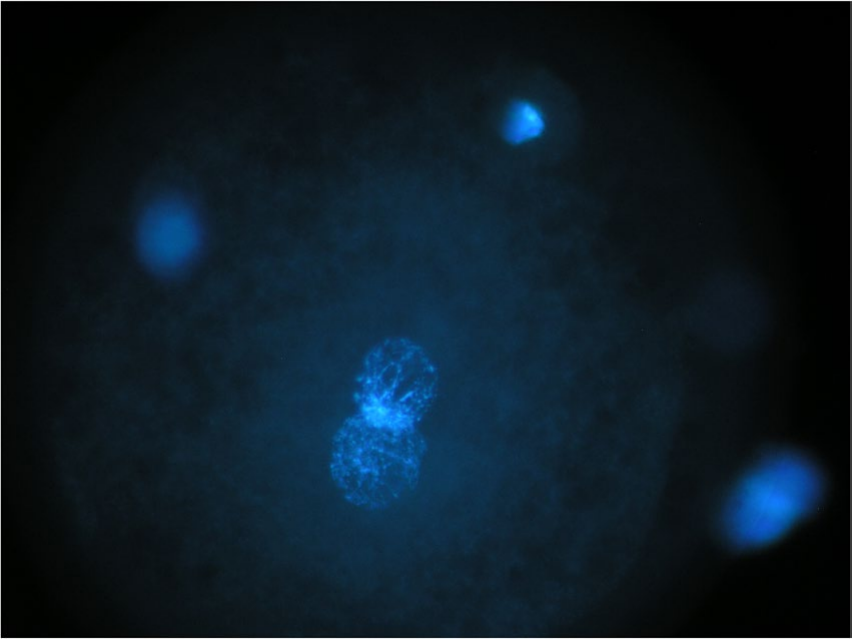
Pronuclear stage evaluated 16 hours after injection with freeze-dried sperm and stained with Hoechst 33342: two polar bodies and two pronucleus.

The nuclear status analyzed after injection and cultured for 16 hours are shown in Table 1. Zygotes were categorized as normally fertilized if one female and one male pronuclei (2 PN) were formed Fig. 3; whereas oocytes with two polar bodies (PBs) and one PN were considered activated without sperm participation. There were no significant differences in term of fertilization rate among the different freeze-dried groups studied, and among freeze-dried sperm groups and frozen thawed control group.

**Table 1:** Female and male pronucleus formation in *in vitro* matured oocytes after injection with frozen-thawed and freeze-dried sperm.

Group	N° Oocytes examined	% of oocyte with 2 PB_S_ and 2 PN_S_	% of oocytes with 2 PB_S_ and 1 PN
Frozen-thawed	30	64.52	12.30
EGTA	30	57.10	9.63
EGTAR	30	61.44	10.26

*PB: polar body; PN, pronucleus*.

**Figure 4:**
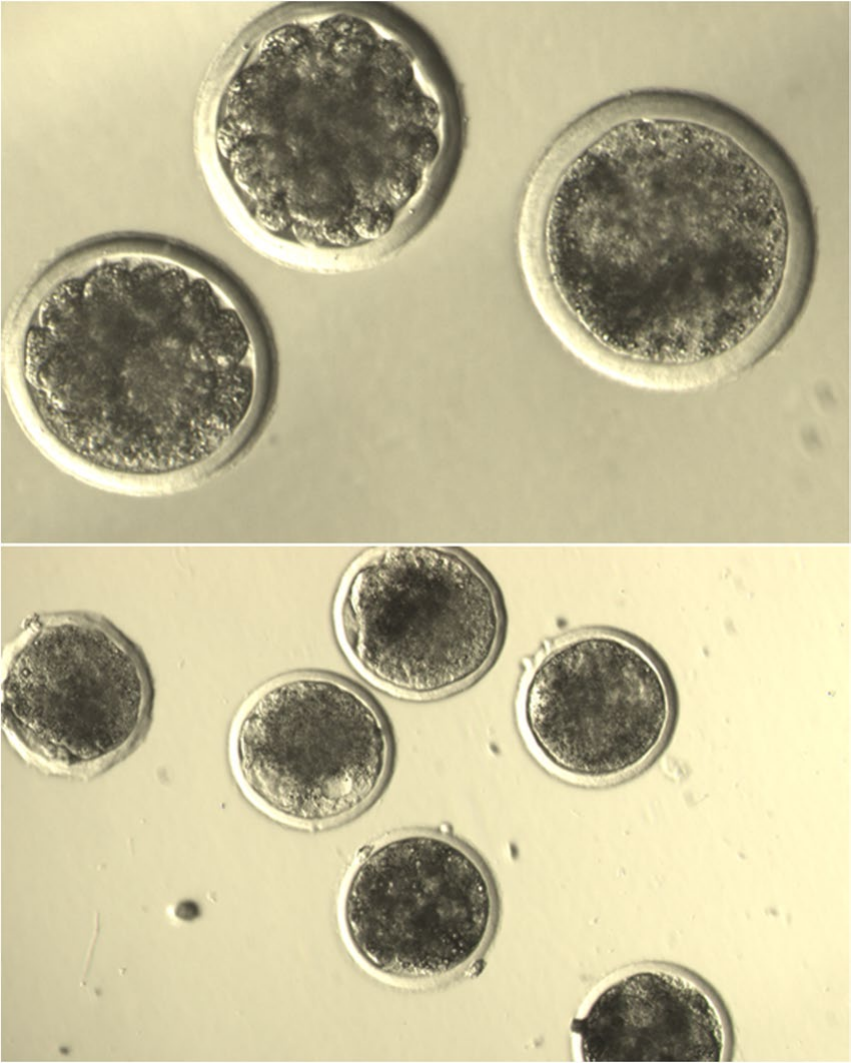
Ovine embryos developed from oocytes 168 h after injection with freeze-dried sperm.

Results of embryonic development from the two groups of freeze-dried sperm (EGTA and EGTAR) and frozen-thawed sperm are shown in Table 2. There were no significant differences among all groups studied on the cleavage rate evaluated 48 h post ICSI. The rate of development to the blastocyst stage was similar for the frozen-thawed control group and freeze-dried group, but superior to parthenogenetic control (sham injection). In addition, no differences were observed between the two freeze-dried sperm groups related to the blastocyst formation rate. Representative embryos resulting from ICSI are shown in Fig. 4.

**Table 2:** *In vitro* development of ovine oocytes after injection with fresh or freeze-dried sperm.

Group	N° Oocytes examined	Cleavage (%)	Embryo development (%)
Frozen-thawed	49	34.7	24.5
EGTA	101	36.6	25.6
EGTAR	87	36.8	24.5
Sham injection	53	30.2	9.4

## Discussion

The freeze-drying technique has recently been applied as a novel method to maintain animal sperm samples. It is expected to be a future tool in animal reproduction technology in order to improve biobanking and storage of genetic diversity^26^.

The present study has demonstrated for the first time that highly effective ovine fertilization and blastocyst development were pursued by using freeze-dried ram spermatozoa and ICSI. A significant proportion of oocytes reached the blastocyst stage when they were injected with freeze-dried spermatozoa. Previous studies using boar sperm suggested that activation and development of oocytes impaired significantly when they were injected with freeze-dried spermatozoa, in comparison with those produced with fresh spermatozoa^9, 27^. However, in this study a similar proportion of blastocysts was obtained when the oocytes were injected with either frozen-thawed or with freeze-dried ram sperm. Kwon *et al*.^9^ reported that substances from sperm heads responsible for oocyte activation are inhibited or inactivated during the freeze-drying processes. In the present study EGTA was added to the freeze-dying extender and no differences were observed between control group (frozen-thawed) and freeze-dried groups in terms of blastocyst formation rate. These results can be explained by the fact that chelating agent inactivates sperm DNase and protects sperm DNA from against disruption during the freeze-drying steps and the subsequent storage^10^. Therefore, chromosomal damage and further negative impact on embryo development can be avoided by using EGTA on freeze-dried spermatozoa.

Another potential source of damage with negative consequences on the quality of embryonic development is the oxidative damage of sperm DNA^12^. Oxidative stress is created when the cellular generation of ROS overwhelms the antioxidant defenses^27^, specially detected during freezing, freeze-drying and rehydration processes. During the cryopreservation, reactive oxygen species promote DNA fragmentation^28^; this is the reason by which the addition of antioxidants to sperm extenders became one of the most suitable alternatives to improve the viability of cryopreserved spermatozoa in different mammalian species such as bull^29^, boar^30^ and ram^31^. It was already suggested that protection of sperm DNA is one of the most important factors for the correct development of oocytes after fertilization; although spermatozoa with fragmented DNA are able to fertilize oocytes, the rate and quality of pregnancy rates decreased significantly^32, 33^. Several studies^16–19, 34^ have shown the beneficial effect of antioxidant therapy on the oxidative stress of mammalian spermatozoa. The addition of antioxidants has important benefits on the quality and fertility of ram semen after liquid storage at different temperatures^35^. To date, there have not been studies focus on determine the antioxidant effect on freeze dried sperm. We have demonstrated for the first time that the presence of antioxidant in the EGTA solution, which was used to freeze-drying, efficiently preserved the DNA integrity of ovine sperm. Therefore, results of this investigation demonstrated that rosmarinic acid has a protective effect against sperm DNA fragmentation during ram sperm freeze-drying process. Rosmarinic acid is a natural antioxidant with therapeutic effects against oxidative stress on cell *in vitro* ^36, 37^. In previous studies^20^, rosmarinic acid showed a high-efficiency free radical-scavenging effect, and it is an important supplement for boar semen cryopreservation. They found that rosmarinic acid supplementation improved the post-thaw quality of boar spermatozoa and the ability to fertilize oocytes *in vitro*. In the present study, freeze-dried semen samples in a medium supplemented with 105 µM rosmarinic acid showed the best results in terms of sperm DNA integrity after rehydration. However, fertilization rate and embryonic development did not differ between oocytes injected with sperm freeze-dried in the presence of rosmarinic acid and those samples lacking this compound. It is well known that some DNA repair factors exist in the oocyte^38, 39^, and fertilized oocytes have the capacity to solve DNA damaging processes^40^. It is true that certain levels of sperm DNA fragmentation have no impact on the fertilization rate and do not compromise blastocyst yield after ICSI^41^. However, when the DNA damage is recognized as irreparable, embryos are excluded by cell cycle arrests or activation of apoptotic pathways^42^. Moreover, *in vitro* culture of oocytes and embryos may lead to dysregulation of many genes^43–45^ resulting in low cellular viability and long-term embryo viability because of a worse competence for DNA damage repair. This repair capacity mainly depends on the length of sperm DNA fragmentation and the cytoplasmic quality of the oocyte. Single-strand breaks (SSBs) could be quickly repaired by oocytes after fertilization, but double-strand breaks (DSBs) could be responsible for chromosome aberrations and loss of genetic materials. Thus, the repair of DSBs in oocytes is more difficult than that of SSBs^46^.

In this study, rosmarinic acid did not increase cleavage rates *in vitro*, although rosmarinic acid-treated sperm exhibited a lower degree of DNA fragmentation. It has been indicated that fertilization with sperm exposed to a DNA damaging agent alters the expression of DNA repair genes at the one-cell stage rat preimplantation-embryo^47^. It is actually possible that the increase in DNA damage on the sperm group without rosmarinic acid leads to an increase in the expression of DNA repair genes in those oocytes injected with spermatozoa from that group.

Men *et al*.^27^ showed that trehalose performed a protective effect against sperm DNA fragmentation on freeze-dried boar sperm, but fertilization and embryonic development were not improved. That study included the analysis of the expression profile of some DNA repair genes in porcine oocytes after intra-cytoplasmic sperm injection^7^ and they concluded that expression of DNA repair genes in fertilized oocytes after ICSI using fresh or freeze-dried sperm, in the presence or in the absence of trehalose, was not different. However, the results of our present study indicated the possibility that the ability of oocytes for DNA repair might neutralize the differences in the level of sperm DNA damage between the two experimental groups. It has been reported that sperm DNA damage levels are directly related to embryonic cleavage rates, so that the activation of additional DNA repair pathways forces delays in cell division^48–50^ resulting in a poor quality blastocyst^51^.

In the present study, we have generated novel data regarding the influence of the storage temperature on freeze-dried sperm samples. We proved that freeze-dried ram spermatozoa can maintain their DNA integrity for long-term storage at room temperature. The first report regarding storage temperature on freeze-dried mouse sperm showed that the fertilizing ability of sperm was impaired when samples were stored at 25 °C^52^. In addition, freeze-dried rat spermatozoa stored at 4 °C for 1 year exhibited lower frequent chromosomal abnormalities than those stored at 25 °C^53^. Several reports indicated that preservation at a low temperature is important for long-term stable preservation of freeze-dried spermatozoa, being 4 °C the most optimal temperature for the preservation of freeze-dried spermatozoa^54, 55^. One of the main goals of freeze-drying studies is the long term storage and simple shipment of spermatozoa at ambient temperature. However, for freeze-dried spermatozoa subjected to the present procedure, long-term storage at 25 °C has not been achieved yet in any mammalian specie. Kwon *et al*.^9^ observed the development to the blastocyst stage only in those oocytes injected with freeze-dried boar sperm stored at 4 °C and not in those injected with freeze-dried sperm stored at 25 °C. On the other hand, normal offspring obtained from oocytes fertilized with freeze-dried mouse spermatozoa that were air-transported at ambient temperatures from Japan to the USA was obtained^56, 57^. In the present study freeze-dried semen samples were stored at 25 °C for one year, leading to the obtention of normal fertilization and blastocyst formation after that sperm was injected in oocytes.

In conclusion, our data confirm for the first time that ovine spermatozoa can be effectively lyophilized, long-term stored at room temperature and successful for embryo development after injected into oocytes. We also demonstrated that the addition of rosmarinic acid to the medium improves sperm DNA integrity after freeze-drying procedures.

## Methods

### Chemicals and media

Unless otherwise stated, all chemicals were from Sigma-Aldrich Co (Alcobendas, Madrid, Spain). The freeze-drying media tested were: EGTA: 10 mM Tris-HCl buffer, 50 mM NaCl and 50 mM EGTA; and EGTAR: EGTA supplemented with 105 µM rosmrinic acid^20^. The final pH of both solutions was adjusted to 8.0–8.2.

The medium used for the collection and washing of cumulus oocyte complexes (COCs) was Dulbeco’s phosphate buffered saline (DPBS) composed of 136.89 mM NaCl, 2.68 mM KCl, 8.1 mM Na_2_HPO_4_ and 1.46 mM CaCl_2_2H_2_O, and supplemented with heparin (50 IU/ml).

The medium used for oocyte maturation was TCM199 supplemented with sheep serum (SS 10% v/v), FSH (0.01 IU/ml), LH (0.02 IU/ml), sodium pyruvate (0.2 mM), estradiol-17b (1 µg/ml), glutamine (2 mM), cysteamine (0.02 mg/ml) and penicillin-streptomycin (0.05 mg/ml). The medium osmolarity was adjusted to 275 mOsm. ICSI was performed in 10 µl droplets of SOF (Synthetic Oviduct Fluid) supplemented with 0.4% BSA and 20 mM of Hepes (BHSOF) under mineral oil. The medium used for *in vitro* culture of sperm-injected oocytes was SOF supplemented with 2% (v/v) BME-essential amino acids, 1% (v/v) MEM-nonessential amino acids, 5% (v/v) Fetal Bovine Serum, 1 mM glutamine, 8 mg/mL BSA and 40 µg/ml gentamicin.

### Sperm collection and freeze-drying procedures

The study was performed following approval by the Veterinary Ethical Committee of University of Zaragoza. The care and use of animals were performed according to the Spanish Policy for Animal Protection RD1201/05, which meets the European Union Directive 86/609 on the protection of animals used for experimental and other scientific purposes.

Sperm was collected from three mature Rasa Aragonesa rams, in the breeding season (October–December) in a twice a week seminal collection regime using an artificial vagina.

Semen quality was previously tested in our laboratory, selecting samples in which all sperm parameters were in the normal ranges for ram semen (motility >85%; total morphology abnormalities <15%).

In each replica, ejaculates from three rams were pooled and divided into three equal fractions including control (cryopreserved) and two test groups (freeze-dried). Fraction to freeze was suspended in Andromed® extender to reach a final semen concentration of 100 × 10^6^ and cooled to 4 °C in 2 h. Then semen samples were packed in 0.25 ml plastic straws and placed 4 cm above the level of liquid nitrogen for 10 min. Finally straws were plunged into liquid nitrogen to store at −196 °C. The fractions to freeze-dry were suspended in the freeze-drying media EGTA or EGTAR to a concentration of 150 × 10^6^/mL and kept at 37 °C for 10 min. 150 μl of sperm suspension from each group was placed into individual cryo tubes (Labcon North America, USA) and plunged into liquid nitrogen for 5 min. Then samples were transferred to a programmable freeze-dryer (LyoBeta 25, Telstar, USA) previously cooled to −50 °C. Samples from the two groups were submitted to a freeze-dying process under the same conditions: a primary drying at a pressure of 0.053 mbar and temperature of −68 °C, and a secondary drying at a pressure of 0.018 mbar and a temperature of 20 °C. Finally, the vials were stored at two different temperatures: 4 °C and at room temperature, for 1 year until used for DNA evaluation and ICSI.

### Sperm preparation and DNA fragmentation analysis

Frozen samples were thawed in a water bath at 37 °C during 21 s for DNA fragmentation analysis. Freeze-dried sperm samples were rehydrated by adding 150 μl Milli-Q water. The sperm suspension was centrifuged for 2 min at 600 g and the supernatant was removed. The sperm pellet was resuspended in 500 μl phosphate buffered saline (PBS) for DNA fragmentation analysis.

Sperm from different treatments were evaluated by the Sperm Chromatin Dispersion test (SCD) specifically designed for ram spermatozoa (Ovis**-**Halomax^®^). The DNA fragmentation analysis in all groups was performed following the manufacturer’s instructions. In brief, the lysis solution was placed at room temperature (22 °C). Then, an eppendorf tube containing agarose was placed in a water bath at 95 °C–100 °C for five minutes, and then transferred in a water bath at 37 °C for five minutes. Meanwhile, 25 μl of each diluted sperm sample was added to an empty eppendorf tube, and 50 ml of liquefied agarose was then transferred into the tube and gently mixed. The temperature of the tubes was maintained at 37 °C. Then, a drop of 2 μl of the cell suspension was placed onto marked wells and each drop was covered with a 24 × 24 mm glass coverslip. The slides were held in a horizontal position throughout the entire process. The slides were placed on a cold surface precooled at 4 °C in a fridge to solidify the agarose. After 5 min the slides were taken out of the fridge and the coverslips were gently removed. Then, the slides were fully immersed horizontally in 10 ml of lysis solution for five minutes. Subsequently, the preparation was introduced into a bath of distilled water for 5 min and then dehydrated by immersion in 2 successive baths of ethanol at 70% and 100% for 2 min each. Finally, the slides were allowed to air-dry before staining. All the slides were stained using a commercial kit for green fluorescence staining (Fluogreen, Halotech DNA SL, Spain). 2 μl of green fluorochrome and mountain medium (1:1; vol/vol) was placed into the well of slide for fluorescent staining of sperm chromatin. The samples were evaluated using fluorescent microscopy (Olympus BX-40, Olympus U-RFL-T, Tokyo, Japan) at magnification 400X and a minimum of 300 spermatozoa were counted per semen sample. Five replicated trials were carried out for each group. Sperm showing a small and compacted halo around a compacted nuclear core contained intact DNA and sperm that displayed a large and spotty halo around the nuclear core corresponded to those sperm with fragmented DNA.

### Oocyte collection and *in vitro* maturation

Oocytes were collected from ovaries of adult sheep at a local slaughterhouse and transported to the laboratory at 35 °C in PBS medium within 1 h. After washing three times the ovaries with saline solution, follicles 2–6 mm in diameter were aspirated. COCs were selected and placed into 50 µl droplets of maturation medium for 24 h at 38.5 °C in a humidified 5% CO_2_ atmosphere under mineral oil. After maturation, oocytes were freed from cumulus cells by gentle aspiration with a pipette in maturation medium supplemented with 0.1% (w/v) hyaluronidase. Denuded oocytes with the first polar body (PB) were harvested under a stereomicroscope and used for ICSI.

### Intracytoplasmic sperm injection

After rehydration or thawed, sperm samples were suspended in BHSOF medium and kept at room temperature for no longer than 2 hours during ICSI. Microinjection was performed at 37 °C with the micro injector (Integra TI). The cumulus-free oocytes were placed in 10 µl drops of BHSOF medium on the cover of a plastic dish. 2 µl of sperm suspension was transferred to a drop of 5 µl of 7% polyvinylpyrrolidone, which was prepared close to the drops containing the oocytes. All drops were covered with paraffin oil. A single sperm in the suspension was aspirated from its tail into the injection pipette, and the pipette was moved to the drop containing the oocyte. The sperm was injected into the ooplasm. The first polar body was either in the 6 or 12 o’clock position, and the injection pipette was in the 3 o’clock position. During the injection, cytoplasm was aspirated to approve that the oolema was broken. This process was repeated until all the oocytes had been injected. The parthenogenetic control was performed by sham injections in a similar manner. And as control group, oocytes were injected with frozen-thawed sperm. Injected oocytes were washed three times with BHSOF and recovered in this medium before activation (5% CO_2_ in air at 38.5 °C). Within 1 h after injection, the oocytes in all groups were activated by adding 5 μM of Ca ionophore for 5 min. After activation, the sperm-injected oocytes were washed three times and *in-vitro* cultured for 6 days (5% CO_2_ in air at 38.5 °C). 16 h after ICSI putative zygotes were fixed for 30 min (0.5% glutaraldehyde in DPBS), stained for 15 min (1% Hoechst 33342 in DPBS), washed in DPBS and examined under an epifluorescence microscope to evaluate the nuclear stage. The criteria used for defining fertilization versus parthenogenetic activation was the presence of two polar bodies and both female and male pronuclei. The cleavage rate and embryo development (compact morulae and blastocyst) were determined at 48 and 144 hours respectively, using an inverted microscope (Nikon Eclipse TE2000-S).

### Statistical analysis

The statistical analysis was performed using SPSS, version 17.0 for Windows. Data concerning DNA fragmentation, pronuclear formation and embryo development were expressed in percentages and analyzed using chi-squared test. Differences were considered significant if P < 0.05.
